# DAHEAN: A Danish nationwide study ensuring quality assurance through real-world data for suspected hereditary anemia patients

**DOI:** 10.1186/s13023-024-03298-4

**Published:** 2024-07-31

**Authors:** Andreas Glenthøj, Andreas Ørslev Rasmussen, Selma Kofoed Bendtsen, Henrik Hasle, Marianne Hoffmann, Klaus Rieneck, Morten Hanefeld Dziegiel, Lene Dissing Sjö, Henrik Frederiksen, Dennis Lund Hansen, Daniel El Fassi, Mathias Rathe, Peter-Diedrich Matthias Jensen, Anne Winther-Larsen, Christian Nielsen, Marianne Olsen, Nina Toft, Mads Okkels Birk Lorenzen, Lise Heilmann Jensen, Sif Gudbrandsdottir, Jens Helby, Maria Rossing, Richard van Wijk, Jesper Petersen

**Affiliations:** 1 Danish Red Blood Cell Center, Department of Hematology, Copenhagen University Hospital - Rigshospitalet, Copenhagen University Hospital, Rigshospitalet Blegdamsvej 9, Copenhagen,, DK-2100 Denmark; 2 Department of Clinical Medicine, Faculty of Health and Medical Sciences, University of Copenhagen, Copenhagen, Denmark; 3 Center for Genomic Medicine, Copenhagen University Hospital - Rigshospitalet, Copenhagen, Denmark; 4 Department of Pediatrics, Aarhus University Hospital, Aarhus, Denmark; 5 Department of Pediatrics and Adolescent Medicine, Copenhagen University Hospital, Rigshospitalet, Copenhagen, Denmark; 6 Department of Clinical Immunology, Copenhagen University Hospital - Rigshospitalet, Copenhagen, Denmark; 7 Department of Pathology, Copenhagen University Hospital - Rigshospitalet, Copenhagen, Denmark; 8 Department of Hematology, Odense University Hospital, Odense, Denmark; 9 Department of Clinical Research, University of Southern Denmark, Odense, Denmark; 10 Hans Christian Andersen Children’s Hospital, Odense University Hospital, Odense, Denmark; 11 Department of Hematology, Aalborg University Hospital, Aalborg, Denmark; 12 Department of Clinical Biochemistry, Aarhus University Hospital, Aarhus, Denmark; 13 Department of Clinical Immunology, Odense University Hospital, Odense, Denmark; 14 Department of Pediatrics and Adolescent Medicine, Aalborg University Hospital, Aalborg, Denmark; 15 Department of Hematology, Aarhus University Hospital, Aarhus, Denmark; 16 Department of Pediatrics, Zealand University Hospital, Roskilde, Denmark; 17 Department of Hematology, Region Zealand University, Roskilde Hospital, Roskilde, Denmark; 18 Central Diagnostic Laboratory, University Medical Center Utrecht, Utrecht University, Utrecht, The Netherlands

**Keywords:** Hereditary anemia, Hemoglobinopathies, Membranopathies, Enzymopathies, Whole genome sequencing, Precision diagnostics

## Abstract

**Background:**

Hereditary anemias are a group of genetic diseases prevalent worldwide and pose a significant health burden on patients and societies. The clinical phenotype of hereditary anemias varies from compensated hemolysis to life-threatening anemia. They can be roughly categorized into three broad categories: hemoglobinopathies, membranopathies, and enzymopathies. Traditional therapeutic approaches like blood transfusions, iron chelation, and splenectomy are witnessing a paradigm shift with the advent of targeted treatments. However, access to these treatments remains limited due to lacking or imprecise diagnoses. The primary objective of the study is to establish accurate diagnoses for patients with hereditary anemias, enabling optimal management. As a secondary objective, the study aims to enhance our diagnostic capabilities.

**Results:**

The DAHEAN study is a nationwide cohort study that collects advanced phenotypic and genotypic data from patients suspected of having hereditary anemias from all pediatric and hematological departments in Denmark. The study deliberates monthly by a multidisciplinary anemia board involving experts from across Denmark. So far, fifty-seven patients have been thoroughly evaluated, and several have been given diagnoses not before seen in Denmark.

**Conclusions:**

The DAHEAN study and infrastructure harness recent advancements in diagnostic tools to offer precise diagnoses and improved management strategies for patients with hereditary anemias.

## Introduction

Globally, more than 7% of the world’s population carries a hereditary anemia variant making them the most prevalent genetic diseases [1] and therefore also responsible for a huge disease burden [2]. Most of these individuals suffer from hemoglobinopathies such as thalassemias and sickle cell disease, which are endemic in areas with a historic high prevalence of malaria [3]. Among Caucasians, hereditary spherocytosis (HS) is the most frequent cause of hereditary anemias [4]. Severe cases of hereditary anemias become evident from early childhood and often require lifelong treatment. Traditional treatments include blood transfusions, iron chelation, and splenectomy [5–7], but an increasing number of novel drugs have demonstrated clinical efficacy [8, 9], with many more currently in clinical trials.

The Danish healthcare system operates on a publicly funded model, financed through general taxation. This framework ensures equal and universal healthcare access for its approximately 5.9 million residents. The healthcare infrastructure is organized into five regions, facilitating localized administration and services. A notable degree of centralization exists under the Danish Health Authority’s guidance. In the context of hereditary anemias, this centralization often limits the authorization to diagnose or treat these conditions to 2–4 hospitals, depending on the subtype. This approach enhances specialized care but also underscores the necessity of ensuring such expertise benefits patients nationwide. The Danish Red Blood Cell (RBC) Center, situated in Copenhagen, aims to act as a comprehensive hub for diagnosis, treatment, and research of RBC diseases. As this manuscript details, it plays a crucial role in coordinating national efforts, bringing together specialists from across Denmark on a monthly basis to collaborate and ensure widespread access to expert care.

### Traditional diagnosis of hereditary anemia in the danish population

Denmark, mirroring global trends, has seen a significant evolution in the diagnostic tools available for hereditary anemias. Detailed clinical history and physical examination remain indispensable in initiating the diagnostic process. Yet, the methodological landscape for diagnosing hereditary anemias has transformed remarkably. Presently, the diagnostic approach integrates various labor-intensive techniques, such as flow cytometry, evaluation of blood smear and bone marrow, electrophoresis, and single gene testing.

Historically, the application of these techniques in a sequential and fragmentary manner often resulted in protracted diagnostic journeys. The inherent complexity of hereditary anemias, especially with the commonality of co-inheriting multiple hemolytic variants, poses substantial diagnostic hurdles. These complexities often manifest as a diverse array of clinical symptoms and were prone to underdetection when relying solely on standard diagnostic workflows [10]. Comprehensive genetic panels were traditionally reserved for elusive cases, and the potential of multi-omics data in unraveling hereditary anemias remained untapped.

### Implementation of tools to enhanced diagnosis of hereditary anemia

The diagnostic landscape is rapidly expanding beyond basic diagnoses such as HS, owing to technological advances [11–14]. Increasingly, we find co-inheritance of multiple genetic variants that in conjunction explain the phenotypic variance [10, 14, 15]. Also, hereditary hemolytic entities are more easily detected by next-generation sequencing (NGS) gene panels [15]. In March 2022, whole genome sequencing (WGS) for hereditary anemias was launched by the Danish National Genome Center and is freely available to patients residing in Denmark. Overall, these technical advances have led to a significant increase in the number of patients diagnosed with hereditary stomatocytosis (HSt) [16] and congenital dyserythropoietic anemias (CDAs), conditions normally considered very rare. While these advances are exciting, genetics alone is often insufficient to provide an accurate diagnosis. Some gene variants display significant variable penetrance, making the impact of specific variants challenging to determine. Consequently, RBC laboratories often struggle to define the pathogenicity of these variants, leading to frequent misinterpretation. To meet this challenge, a strengthening of highly specialized diagnostic frameworks is strongly warranted [17]. Recent initiatives in Denmark aim to integrate high-throughput proteomics and automated patch-clamp measurements into routine diagnostics, enhancing the precision and efficiency of diagnosing hereditary anemias. As we move forward, these efforts are expected to be complemented by the inclusion of multi-omics data, offering a holistic view of the genetic, proteomic, and metabolic landscapes underlying these complex disorders.

The Danish Hereditary Anemia (DAHEAN) study aims to consolidate diagnostic and clinical capacities across Denmark to offer patients access to such advanced diagnostics and thereby facilitating optimal management, including participation in clinical trials. Furthermore, the study serves as a framework for improvement of diagnostic tools and identifying patients with ultra-rare disease that could be eligible for interventional clinical trials.

## Materials and methods

### Design

**Fig. 1:**
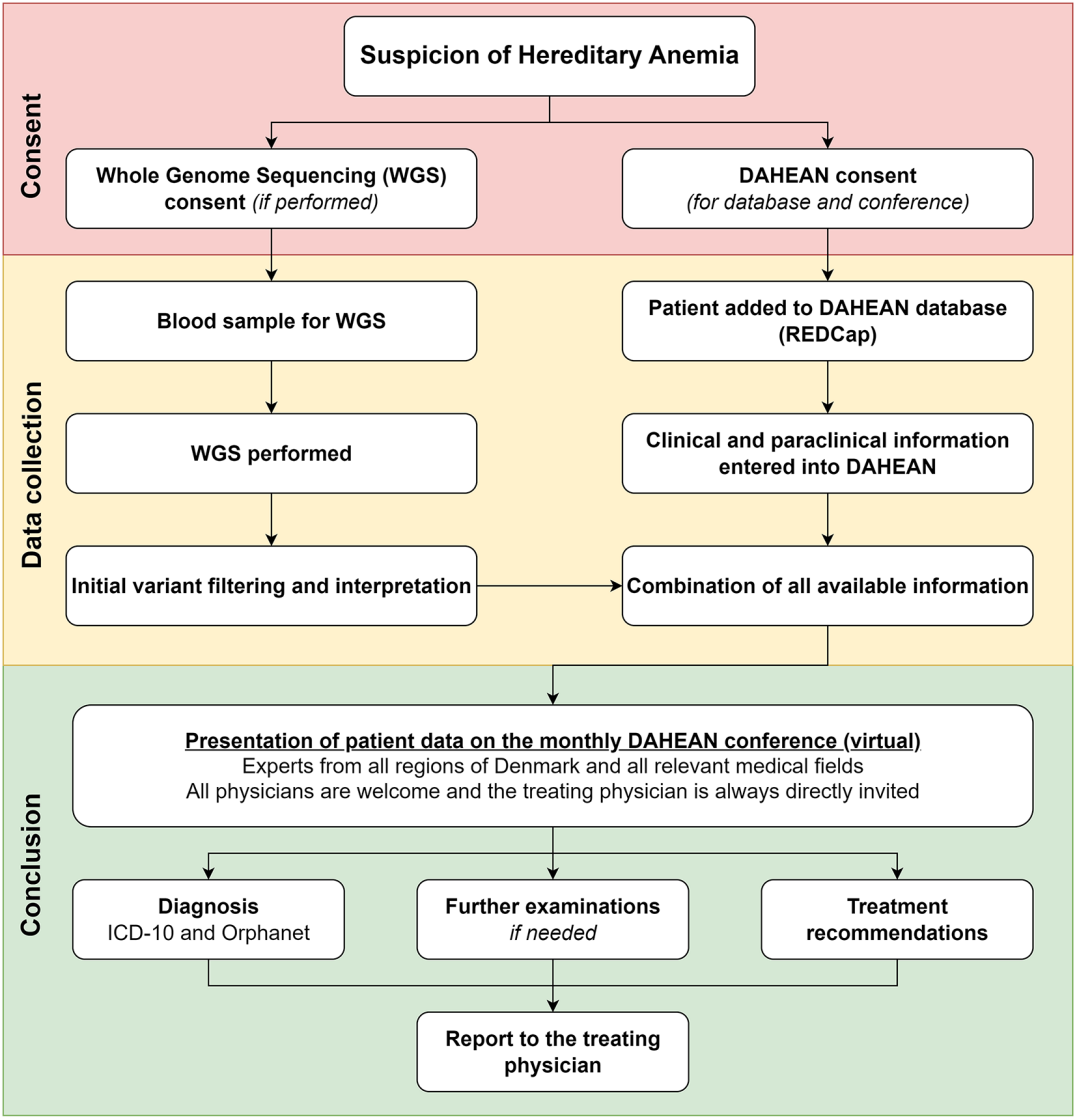
Flowchart of the Danish Hereditary Anemia (DAHEAN) study. Any patient suspected of or diagnosed with hereditary anemia may be included. Consent is necessary for participation. Whole genome sequencing (WGS) may be performed if clinically indicated. When necessary clinical and paraclinical data has been gathered, patients are discussed at the monthly national anemia boards, which provides diagnostic and therapeutic recommendations to the treating physician

Prospective cohort study of patients residing in Denmark with a suspected or already diagnosed hereditary anemia (Fig. 1). The study was approval by the Ethical Committee of the Capital Region of Denmark (H-21064560) and the Danish Data Protection Agency (P-2021-736). Patients provided informed consent to participate.

Patients were included from participating clinical departments (Table 1). Patients with existing hereditary anemia diagnoses were included at the physician’s discretion to validate, refine, or change previous diagnoses using WGS, identify additional genetic variants affecting disease management, and enhance understanding of hereditary anemias. This approach aims to open new avenues for treatment and care by integrating advanced genetic insights. Diagnostic procedures were only performed when relevant and consequently, not all analyses are available on all patients.

**Table 1:** Participating departments

Departments	Age group
**Capital region of denmark**	
Department of Hematology, Copenhagen University Hospital - Rigshospitalet	Adults
Department of Pediatrics and Adolescent Medicine, Copenhagen University Hospital - Rigshospitalet	Children
**Central Region Denmark**	
Department of Hematology, Aarhus University Hospital, Aarhus	Adults
Department of Pediatrics, Aarhus University Hospital, Aarhus	Children
**Region of Southern Denmark**	
Department of Hematology, Odense University Hospital, Odense	Adults
Hans Christian Andersen Children’s Hospital, Odense University Hospital, Odense	Children
**Northern Denmark Region**	
Department of Hematology, Aalborg University Hospital, Aalborg	Adults
Department of Pediatrics and Adolescent Medicine, Aalborg University Hospital, Aalborg	Children
**Region Zealand**	
Department of Hematology, Region Zealand University, Roskilde Hospital, Roskilde	Adults
Department of Pediatrics, Zealand University Hospital, Roskilde	Children

### Data capture

Patient data were collected using REDCap (Research Electronic Data Capture) electronic data capture tools hosted by the Capital Region of Denmark [18, 19]. REDCap is a secure, web-based software platform designed to support data capture for research. Each department were allocated to a data access group in which they can only access their own patients. Only administrators have access to patient information across sites.

WGS raw data were stored by Danish National Genome Center (NGC; https://eng.ngc.dk*)* according to standard operating procedures.

### Incidental genetic findings

Pseudopanels were used for extensive genetic testing, which minimized the risk of detecting pathogenic germline variants unrelated to anemia in patients. If such variants were detected, patients were offered a referral to the genetic counseling services at their local Department of Clinical Genetics.

### Study subjects

Patients were recruited from hematological, pediatric, and clinical genetic departments and offered inclusion through their local physician. Inclusion criteria were broad: (1) All ages, (2) Signed informed consent from patient or their legal guardian (consents currently available in Danish and English), (3) Suspicion or verified diagnosis of a hereditary anemia. There were no exclusion criteria. Criteria were intentionally left very broad to facilitate that any patients that might benefit from comprehensive evaluation for hereditary anemia could be included. Consequently, cohort composition very much depended on the referring physician’s preference, which will likely also develop over time as physicians – hopefully – experience benefit of including patients.

As pathogenetic *PIEZO1* mutations can cause a membranopathy - patients evaluated genetically for JAK2-wildtype erythrocytosis were included in the cohort. Additionally, genetic disorders of iron metabolism may also impair erythropoiesis and patients suspected of these were also eligible for this cohort.

### Clinical information

Relevant clinical information was gathered by the patient’s physician and typed into a secured electronic database (see above). Information included name, social security number, relevant medical history, blood works, blood transfusion data, radiology, family medical history, and ethnic origin. Ethnicity was essential to genetic analysis as frequency of gene polymorphisms vary in different ethnic populations and thus influence the evaluation of genetic variants. Data were, whenever possible, captured in a structured manner using standardized tools such as human phenotype ontology (https://hpo.jax.org*)* and SNOMED (www.snomed.org*).*

### Genetic analyses

**Fig. 2:**
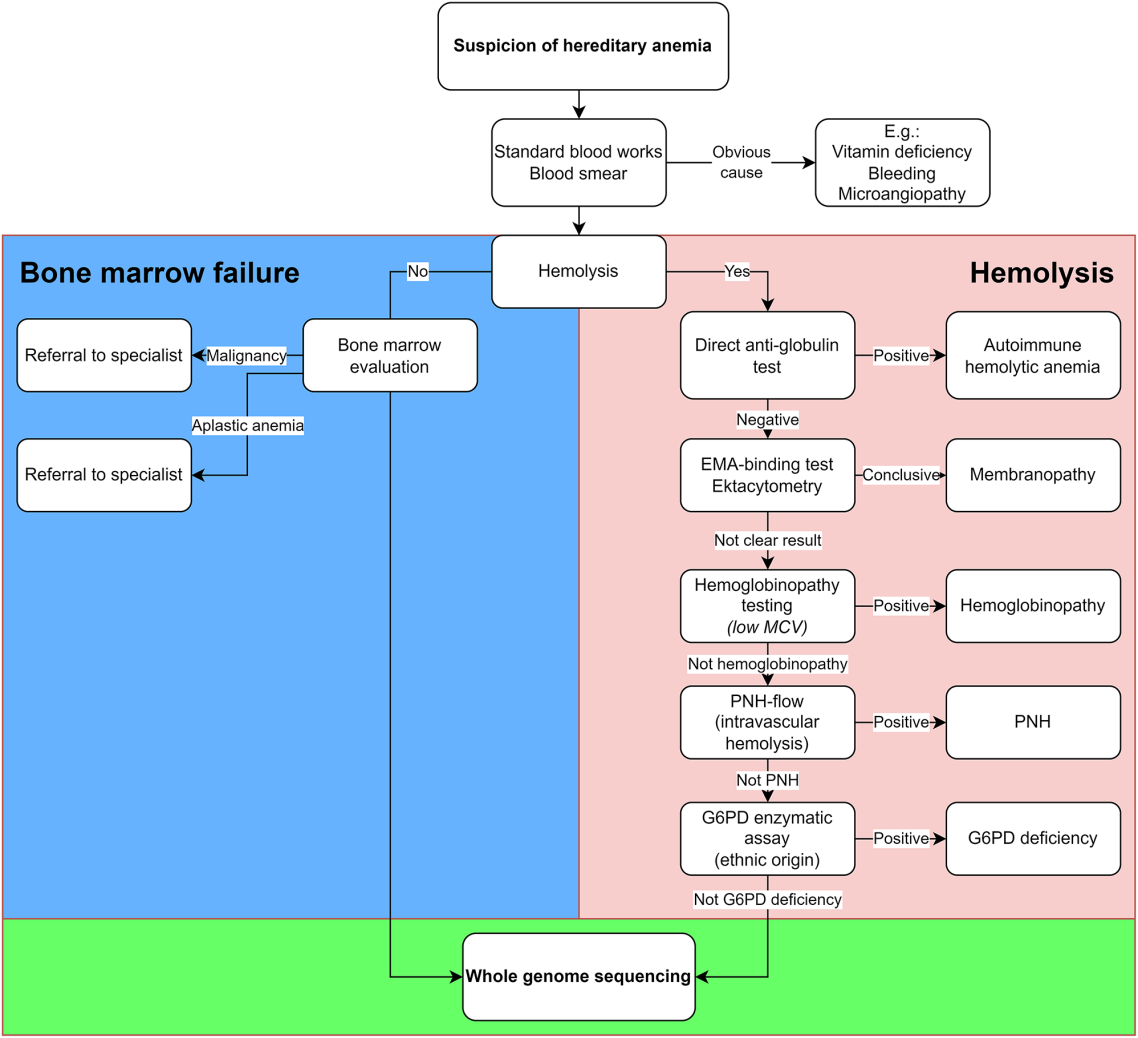
Diagnostic Flowchart for Hereditary Anemia. Structured approach for adding whole-genome sequencing (WGS) to the diagnostic algorithm. diagnosing hereditary anemia. Abbreviations: AIHA: Autoimmune Hemolytic Anemia, DAT: Direct Anti-globulin Test, EMA: Eosin-5-maleimide, G6PD: Glucose-6-Phosphate Dehydrogenase, MCV: Mean Corpuscular Volume, PNH: Paroxysmal Nocturnal Hemoglobinuria, WGS: Whole Genome Sequencing

Whole genome sequencing (WGS) was performed by the NGC according to NGC standard operating procedures. Originally, WGS was envisioned as a last resort for cases of hemolytic anemias that remained unresolved after exhausting the standard diagnostic pathway (Fig. 2). Nonetheless, the adherence to this protocol was not enforced, granting the referring physician the autonomy to decide on the use of WGS based on their clinical judgment.

WGS was done using Illumina PCR-Free library preparation and Illumina platform sequencing. Bioinformatic processing was based on a standardized and continuously updated pipeline. Alignment of sequencing reads to the hg38/GRCh38 reference genome was done using BWA [20]. Single nucleotide variant (SNV) calling was performed with GATK (following best practices) [21]. Structural variant (SV) calling was performed using a combination of several tools: Manta [22], Delly2 [23], Lumpy [24] and CNVnator [25]. Variants were annotated and filtered using VarSeq (Golden Helix, Inc., Bozeman, MT, www.goldenhelix.com). These analyses required consent to comprehensive genetic analyses (available from www.anemia.dk). Although used in most patients, WGS was *not* a prerequisite for participation in the study.

More than 160 genes implicated in hereditary anemias were included in a continuously updated gene panel (Supplementary Table 1), that was used for *in silico* filtering of variants. However, novel anemia associated genes could also be searched for and identified. This especially pertained to patients with a family history of anemia of unknown origin or patients with unusual phenotype. Gene prioritization of anemia associated genes is done using the inbuilt VarSeq algorithm PhoRank.

Variant interpretation and classification was done according to recommendations from the American College of Medical Genetics (ACMG) [26] with inclusion of refinements to the guidelines recommended by Clinical Genome (ClinGen) Resources Sequence Variant interpretation subgroup (https://clinicalgenome.org/working-groups/sequence-variant-interpretation/*)* and relevant ClinGen expert panels. The molecular genetic effect of variants was evaluated using VarSeq and the integrated software Alamut Visual Plus (SOPHiA GENETICS, USA). Classification was based on information from relevant clinical (e.g. NCBI ClinVar, HGMD [27]) and population databases (e.g. gnomAD [28]), extensive searches for prior reports in the medical literature, and *in silico* predictions using REVEL [29] for missense variants and a combination of MaxEntScan [30] and SpliceAI [31] for splice altering variants. Online Mendelian Inheritance in Man (OMIM) nomenclature was used to subtype genetic diseases.

In selected cases, other genetic techniques such as array CGH, Sanger sequencing, and targeted sequencing of specific genes [32] was employed.

### Functional assays

Functional assays were used to diagnose hereditary anemias and to verify the pathogenicity of genetic variants found.

### Protein quantification

#### Mass-spectrometry based proteomics and metabolomics

As a pilot project, blood from selected patients investigated for hereditary anemias were subjected to mass-spectrometry based proteomics at the Department of Clinical Biochemistry at Bispebjerg Hospital [33] and metabolomics at the University Medical Center Utrecht, The Netherlands [34].

### Protein and peptide measurements

Hemoglobin fractions were routinely quantified by high-pressure liquid chromatography (HPLC) [35, 36] at Aarhus University Hospital and Rigshospitalet. Specific protein quantification could be performed by Sodium Dodecyl Sulfate Polyacrylamide Gel Electrophoresis (SDS-PAGE), Western blotting, and Enzyme-Linked Immunosorbent Assay (ELISA). Assays of erythropoiesis and hemolysis including erythroferrone, GDF15, soluble transferrin receptor, and hepcidin were available [37] if relevant. Hemoglobin stability was assessed by isopropanol precipitation test [38].

### Cytology

Blood marrow and peripheral blood were assessed according to standard procedures at the local department of pathology [39]. Central review at the Department of Pathology at Rigshospitalet was often utilized.

### Membranopathies

RBC membrane were studied by standard diagnostic procedures including osmotic gradient ektacytometry (RR Mechatronics) [11, 32, 40] and flow cytometry including Eosin-5-maleimide (EMA)-binding test [32, 36, 40].

Functional tests of RBC ion channels were experimental and lacked standardization. With this cohort, we aim to validate novel assays, including one automated patch-clamp [41].

### Enzymopathies

Enzymatic assays were performed to verify enzyme deficiencies. These included measurement of pyruvate kinase [42] and glucose-6-phosphate dehydrogenase (G6PD) activity. Enzymatic assays unavailable in Denmark were performed at the EuroBloodNet laboratory at the University Medical Center Utrecht, The Netherlands.

### Hemoglobin oxygen affinity

Oxygen affinity of hemoglobin was assessed by local arterial blood lactate (ABL) analyzer or more detailed methods such as HEMOX [43]. For when patients with sickle cell disease are enrolled, Oxygenscan [44] is available for phenotype assessment.

### Autoimmune assays

Autoimmune assays include direct antiglobulin test, cold agglutinin titers, Donath-Landsteiner test. Screening tests were typically performed locally, but more sensitive and specific flowcytometric tests were available at the Department of Clinical Immunology at Rigshospitalet [45].

### Statistical considerations

Due to the exploratory nature of the study, the study did not aim to reach a specific number of patients and a power calculation was therefore not needed. Inclusion of 50–100 patients per year was expected.

### European collaboration

Patients identified with a rare hereditary anemia were offered inclusion in the Rare Anemia Disorders European Epidemiological Platform (RADeep; https://www.radeep.eu*)* to help map prevalence, increase understanding of disease phenotype, and promote translational research in rare anemias.

Patients who remained diagnostically or therapeutically unresolved after a DAHEAN conference could be assessed via the Clinical Patient Management System of ERN-EuroBloodNet (https://cpms.ern-net.eu*)* to provide specialized, expert medical care on a European level.

## Results

### Patient characteristics

**Fig. 3:**
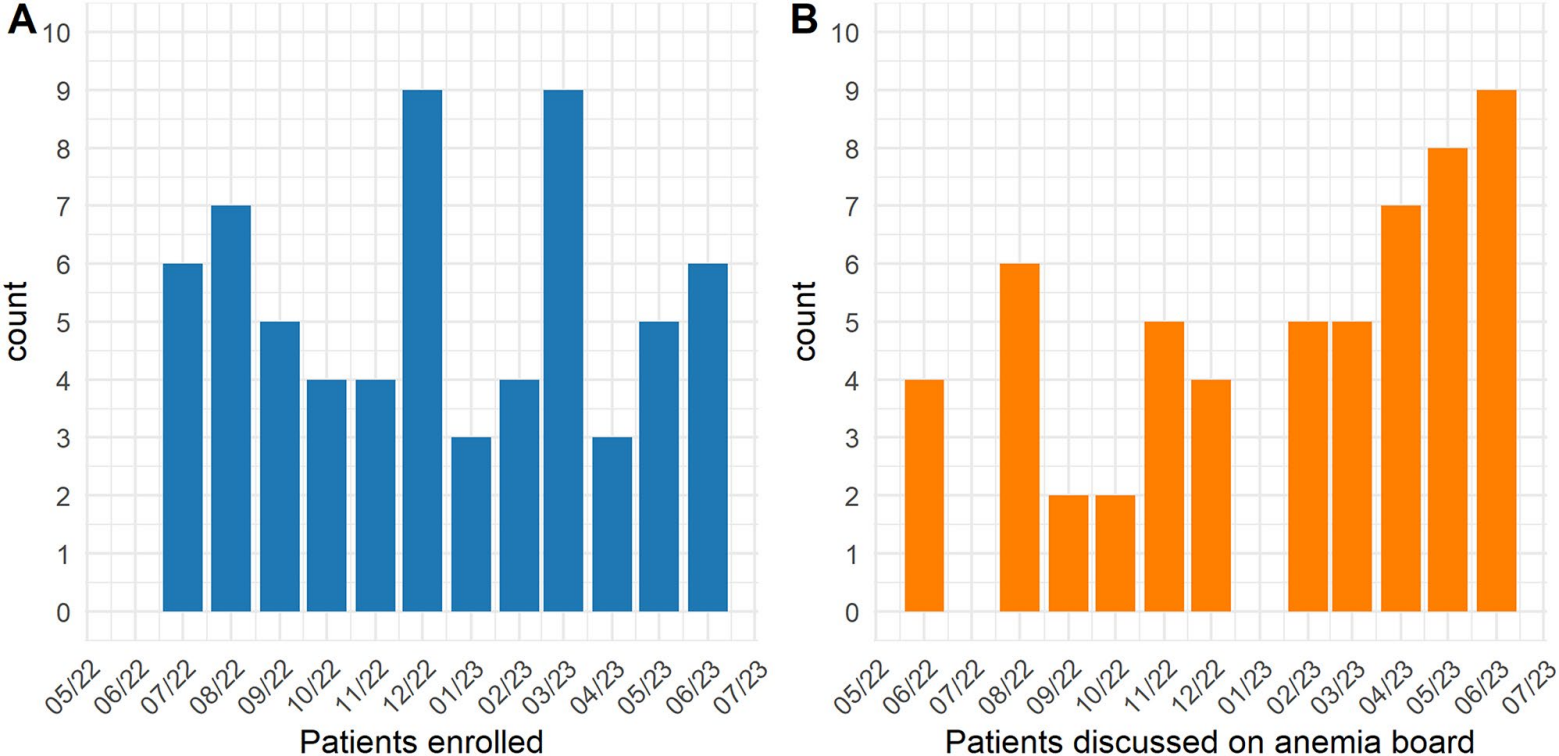
Bar graphs depicting the number of patients enrolled and discussed on anemia board. Anemia boards were typically held on a monthly basis, with the exception of July, due to reduced attendance during the summer holiday period

The DAHEAN registry received approval on May 12th, 2022, and the first patient gave consent on June 20th, 2022. As of the cut-off date of 30th of June 2023, 76 patients had consented to participate in the registry (Fig. 3A). Additionally, some healthy relatives of included patients also consented to the registry and to WGS (e.g., for trio analysis), but these individuals were analyzed in the context of the index patient and results are not included here. The demographics of all patients are presented in Table 2. 55/76 (73.3%) of patients were from the Capital Region of Denmark. The median age was 40 years, sex distribution was equal, and 68/76 (89.5%) were adults.

**Table 2:** Demographic and clinical characteristics of participants

Variable	Category	n%
Total participants	Overall	76
Sex	Female	38 (50.0)
	Male	38 (50.0)
*Age (years)*	*Median (range)*	*40 [1–93]*
Age Group	1–17 years	8 (10.5)
	18 + years	68 (89.5)
Administrative Region of Denmark	Capital	56 (74.6)
	Mid	7 (9.3)
	Northern	4 (5.3)
	Southern	6 (8.0)
	Zealand	2 (2.7)
Transfusions in the Last 12 Months	None	64 (84.2)
	Intermittent	7 (9.2)
	Regular	5 (6.6)
Splenectomy	No	73 (96.1)
	Yes	3 (3.9)
Cholelithiasis	No	55 (74.3)
	Yes	19 (25.7)

Included patients were suspected of or previously diagnosed with a broad range a hereditary RBC disorders, including disorders of iron metabolism and even polycythemia as *PIEZO1* – normally associated with dehydrated hereditary stomatocytosis - has also been described as a frequent cause of familial polycythemia [46]. Paraclinical hemolysis in the context of polycythemia often triggered WGS and inclusion in the study, although such criteria were not mandatory. The inclusion of patients with suspected *PIEZO1* variants aims to deepen our understanding of their potential roles in erythrocytosis and other RBC disorders. This approach enables a broader exploration of genetic contributions to erythrocyte function, enhancing both diagnostic precision and the development of targeted therapies.

Some patients experienced their first symptoms decades before the DAHEAN collaboration was established. New patients presenting with severe symptoms, especially children, could be rushed with WGS results being available in 1–2 weeks.

Only 12/76 (16%) of patients had a history of RBC transfusion within the last year, only 3/76 were splenectomized (4%), whereas 19/76 (26%) had a history of cholelithiasis (Table 2).

### Anemia boards

During the first thirteen months, 11 national anemia boards were held. Median number of participants were 12 (range 9–20) with representation from all five Danish administrative regions. Medical specialties represented included adult and pediatric hematology, clinical immunology, clinical biochemistry, clinical genetics, genomic medicine, and pathology.

As of 30th June 2023, 57/76 (75.0%) of included patients have been discussed at the national anemia board (Fig. 3B), and a report with diagnostic evaluation and therapeutic recommendation has been sent to the treating physician. The remaining patients await evaluation, almost exclusively because of waiting time on whole genome sequencing.

#### Diagnostic efficacy

In 43 out of 57 patients discussed at the national anemia board (75%), a precise diagnosis was established or verified with hereditary spherocytosis as the most common type. (Table 3). Three were diagnosed with hyperbilirubinemia type 1 (Gilberts syndrome) and absence of hemolysis.

**Table 3:** Orphanet diagnoses of participants

ORPHAcode	Name	n%	
**Hemoglobinopathies**			
846	Alpha-thalassemia	1 (1.8)	
848	Beta-thalassemia	1 (1.8)	
99139	Unstable hemoglobin disease	2 (3.5)	
**Enzymopathies**			
362	Glucose-6-phosphate-dehydrogenase deficiency	1 (1.8)	
766	Pyruvate kinase deficiency of erythrocytes	1 (1.8)	
712	Hemolytic anemia due to glucophosphate isomerase deficiency	1 (1.8)	
**Membranopathies**			
288	Hereditary elliptocytosis	1 (1.8)	
822	Hereditary spherocytosis	15 (26.3)	
3202	Dehydrated hereditary stomatocytosis	3 (5.3)	
3203	Overhydrated hereditary stomatocytosis	1 (1.8)	
398088	Hereditary cryohydrocytosis with normal stomatin	1 (1.8)	
**Malignancy**			
98827	Myelodysplastic syndrome	1 (1.8)	
824	Primary myelofibrosis	1 (1.8)	
**Iron Metabolism**			
163	Hereditary hyperferritinemia-cataract syndrome	1 (1.8)	
209981	Iron refractory iron deficiency anemia (IRIDA)	1 (1.8)	
**Other**			
98428	AD secondary erythrocytosis	1 (1.8)	
621	AR methemoglobinemia	1 (1.8)	
98873	Congenital dyserythropoietic anemia type II	3 (5.3)	
357	Hyperbilirubinanemia type 1 as primary diagnosis	3 (5.3)	
2322	Kabuki syndrome	1 (1.8)	
90042	Primary familial polycythemia	1 (1.8)	
98428	Secondary polycythemia	1 (1.8)	
**None**		14 (24.6)	
**Total Participants (Overall)**		**57**	

WGS was performed on 56 of the 57 patients, except one diagnosed with Hb Southampton via Sanger sequencing. The board found that WGS conclusively established a diagnosis in 26 cases and significantly increased diagnostic likelihood in 7 cases. Additionally, in 18 of the other cases, WGS was considered of value to the diagnostic process, for instance, by making certain differential diagnoses unlikely or elucidating the cause of elevated bilirubin levels.

Notably, cases of ultra-rare anemias were identified including the first cases in Denmark of glucophosphate isomerase (GPI) deficiency (*n* = 1), hereditary cryohydrocytosis (*n* = 1) and congenital dyserythropoietic anemia type 2 (CDAII; *n* = 3).

Overall, genetic workup either solidified or altered the treatment approach for 34 patients. However, in 22 cases, WGS had no impact on therapeutic decisions.

## Discussion

The establishment of the DAHEAN network represents a significant consolidation of national expertise, fostering advanced diagnostic processes for patients with suspected hereditary anemias. The implementation of monthly anemia boards successfully brings together experts across several medical specialties at the national level in Denmark, promoting cross-regional collaboration and knowledge exchange. The unique access to free-of-charge WGS fostered an opportunity to evaluate its potential benefit.

However, we observe an uneven inclusion of patients from participating centers. A significant majority (71.4%) of patients originate from coordinating site, Rigshospitalet, situated in the Capital Region, which accounts for only 31.9% of the Danish population. This disproportionate representation was partly due to implementation delays of WGS in some regions and their corresponding centers. Furthermore, individual clinicians, local organization and sub-specialization may have influenced the utilization of anemia diagnostics. In practice, decision of when WGS should be performed was left to the referring physician, which complicates interpreting the precise value of implementing the originally intended diagnostic algorithm (Fig. 2). Conversely, deviations from the original algorithms likely reflected that clinicians gradually experienced cases of added value of a comprehensive genetic and functional evaluation at the anemia boards. Future efforts are needed to enhance the implementation of advanced diagnostic tools like WGS across all regions to ensure more uniform patient representation.

Despite our study’s intentions, a vast majority (over 90%) of the patients were adults. Although WGS is often used in pediatrics, inclusion of pediatric patients was delayed by several practical obstacles such as getting written consent from both guardians or delayed implementation of WGS in some regions and their corresponding pediatric departments. Moreover, the department of (adult) hematology in the Capital Region was the first to open for inclusion of patients and at the same time specializes in rare anemias and consequently follows a disproportionate number of candidates for WGS. Nonetheless, providing a precise molecular diagnosis for young patients is a key future priority as it is the foundation for optimal treatment and future trials in ultra-rare hematological diseases [47]. But for now, the uneven geographical and age distribution makes it difficult to apply the Danish experiences to other settings. To ensure a broader age distribution, initiatives have been implemented to promote the study within pediatric hematology. This includes a new webpage (www.anemia.dk), where information on WGS, anemia boards, and consents have been gathered to facilitate including of patients from all centers. Furthermore, uncertain diagnostic test results are now often supplemented with a recommendation of doing WGS.

One of the key successes of the DAHEAN network is the precise diagnosis of ultra-rare conditions such as GPI deficiency, hereditary cryohydrocytosis, and CDAII, which likely would not have been diagnosed outside a highly specialized setting. CDAII is often mistaken for the far more common HS due to their similarity in initial clinical presentation and results of diagnostic tests such as EMA-binding and osmotic gradient ektacytometry [48]. However, patients with CDAII respond poorly to splenectomy and have long-term risk of non-transfusion-dependent iron overload [49], underscoring the need for correct diagnosis to ensure optimal management. Although bone marrow examination can readily identify CDAII, this test is often not performed. Therefore, the access to WGS proves highly valuable in identifying *SEC23B* variants characteristic of CDAII, and effectively pointing to the correct diagnosis. Such cases form basis for a continuous discussion at the anemia boards on who to offer WGS. As clinicians encountered added diagnostic or therapeutic value of comprehensive genetic evaluation of patients, the algorithm shifted towards a more liberal usage of WGS. However, despite using a genetic panel, broad usage of WGS failed to produce diagnostic or therapeutic results in about 40% of patients and still inferred an inherent risk of incidental findings and need for genetic counselling in these cases.

A significant challenge in employing genetic testing lies in the uncertainty regarding the pathogenicity of identified variants. To address this issue, functional testing has been incorporated into the diagnostic protocols employed by anemia boards. Currently, this integration primarily utilizes established techniques, including enzymatic assays, ektacytometry, and blood cell cytology. However, there are active research initiatives aimed at enhancing these diagnostic capabilities through the development of the DAHEAN scaffold. This framework is designed to facilitate the integration of advanced mass spectrometry proteomics and automated patch clamp techniques into the standard diagnostic repertoire, thereby expanding the toolkit available for the precise identification and characterization of anemias. The utility of such advancements becomes particularly evident in the context of RBC ion-channel disorders, where the genetic heterogeneity of entities like *PIEZO1*, coupled with the suboptimal accuracy of existing assays, often hinders accurate diagnosis. Future studies of this cohort will detail the genetic variants found in context of accompanying validations assays.

## Conclusions

Overall, the DAHEAN network shows the vast potential of national collaboration and advanced diagnostic tools in the identification and management of hereditary anemias. While there are challenges to address, the successes achieved so far at this early stage are encouraging and provide concrete evidence for the feasibility and significant utility of such a collaborative, nationwide approach.

## Data Availability

The data that support the findings of this study are available from the corresponding author upon reasonable request. Due to privacy concerns and to protect the confidentiality of study participants, the data cannot be openly shared.

## Declarations

### Generative AI and AI-assisted technologies in the writing process

During the preparation of this work, the authors used OpenAI’s language model, GPT-4 in order to improve language and readability. After using this tool/service, the authors reviewed and edited the content as needed and take full responsibility for the content of the publication.
